# Digital Capability, Open-Source Use, and Interoperability Standards Within the National Health Service in England: Survey of Health Care Trusts

**DOI:** 10.2196/66398

**Published:** 2025-08-12

**Authors:** Matthew Russell Bennion, Ross Spencer, Roger K Moore, Richard Kenyon

**Affiliations:** 1 School of Computer Science, University of Sheffield, Regent Court, 211 Portobello, Sheffield, S1 4DP, United Kingdom, 44 1142221800; 2 Digital Innovation Unit, NHS Midlands and Lancashire Commissioning Support Unit, Stoke-on-Trent, United Kingdom; 3 Unaffiliated, Leipzig, Germany; 4 Aston Business School, Aston University, Birmingham, United Kingdom

**Keywords:** NHS, healthcare, open source, technologies, digital capability, capabilities, GDE funding, interoperability, surveys, England, Global Digital Exemplar, comparative analysis, document, initiative, Department of Health and Social Care, National Health Service

## Abstract

**Background:**

In 2016, the National Health Service (NHS) England sought to drive digital transformation within select NHS trusts through the Global Digital Exemplar (GDE) program. While the program did advance the NHS’s integration with digital technologies, disparities in digital maturity persisted between GDE-funded and nonfunded NHS trusts. The Department of Health and Social Care (DHSC) launched a data strategy in 2022 that aimed to develop the appropriate technical infrastructure and data architecture to enable more effective and efficient use of its data. Given the diversity in digital capabilities, open-source adoption, and interoperability standards within NHS services, official guidance has continued to struggle to provide effective unification. Data about capabilities and technologies from application development teams in the NHS trusts, crucial for advancing these areas, remains insufficient.

**Objective:**

This study aimed to further document the capabilities and technologies used in the NHS to develop digital capacity, comparing those with standard funding against those with additional GDE funding. This comparative analysis provides a foundational understanding for evaluating current practices and identifying potential areas for improvement in the NHS digital transformation efforts.

**Methods:**

This study was conducted using Freedom of Information (FOI) requests and systematic website searches. The Freedom of Information Act (FOIA) allows individuals to request information held by public authorities. This process supports transparency and accountability by ensuring public access to government data. Data were compiled from two sources: (1) FOI requests submitted to NHS trusts between July 2020 and December 2020, and (2) systematic website searches for technology conducted between August 2020 and July 2021. A series of chi-square tests was conducted to validate and strengthen the robustness of the FOI questions.

**Results:**

A total of 191 (84.5%) of the then 226 NHS trusts completed the FOI request, and 161 of the 191 (84%) had software and app development, website, or innovation teams. A total of 112 (69.6%) teams developed front-facing service user websites and apps. Out of 191, 150 (93.2%) worked with clinical staff to formulate innovative ideas, 55 (34.2%) carried out developments for other trusts and external entities, 35 (21.7%) had attempted to secure an innovation grant, and 138 (86%) disclosed the technologies they use. A total of 25 (15.5%) said they always used open-source technology, and 24 (17%) disclosed technologies associated with interoperability standards in their responses.

**Conclusions:**

The NHS must adopt a cohesive strategy and refine policies to ensure the success of its digital, open-source technology and interoperability standards initiatives. Five recommendations toward greater organizational interoperability are made by the authors. Future research should examine digital innovation across NHS trusts, focusing on barriers such as limited resources, organizational culture, and technical expertise. Identifying these challenges is essential for developing strategies to reduce disparities and promote equal progress.

## Introduction

### Background

In 2022, the Department of Health and Social Care (DHSC) released its data strategy, “Data Saves Lives: Reshaping Health and Social Care with Data,” to improve how the National Health Service (NHS) uses data [1]. A key goal of the strategy was to develop the appropriate technical infrastructure to ensure that the data architecture underpinning the NHS works in unison, enabling more effective and efficient use of data. The data strategy would be carried out via a commitment to develop and publish a draft of the standards alongside an interoperability strategy to ensure that fit-for-purpose standards are widely adopted across health and adult social care. The anticipated standards would reuse and build upon international standards where applicable. However, the NHS’s current ability to fulfill this strategy across hospitals and other facilities is not well understood due to a lack of visibility into its digital development capabilities.

The Global Digital Exemplar (GDE) program was conceived in 2016 by NHS England to achieve digital transformation in select NHS trusts. It was hoped that the program would create a knowledge-sharing ecosystem to spread learning to other NHS trusts [2]. The program resulted in the creation of 16 GDE acute trusts [3,4], 3 GDE ambulance trusts [5,6], and 7 GDE mental health trusts [7].

While the GDE program improved the use of digital within the NHS, studies carried out since have highlighted that NHS trusts and social care providers still lack digital maturity [8], and that a digital divide has emerged between NHS trusts with and without GDE funding [9,10].

The “Data Saves Lives” strategy uses user stories to describe various systems that can work together in a secure, safe, accessible, and interoperable manner. Within this strategy, the UK Health Security Agency (UKHSA) aims to prioritize FAIR (findability, accessibility, interoperability, and reusability) principles [11]. In the context of FAIR, interoperability is defined as “the data need to interoperate with applications or workflows for analysis, storage, and processing.” However, interoperability should not be viewed solely from a data perspective; it must also encompass system interfaces, the methods by which data are produced and consumed across the NHS ecosystem. While a universal approach to the architecture of NHS systems is impractical, achieving interoperability requires a commitment to key design considerations across data and application interfaces. This ensures the secure, safe, and effective use and reuse of data and information across system and organizational boundaries.

To complement interoperability, NHS England’s Transformation Directorate is also in pursuit of making source code within the NHS freely available and downloadable through the use of open-source. Both open-source and interoperability can save NHS services from duplicating effort and help them build better services faster through shared resources [12]. The NHS Transformation Directorate presently promotes the open-source languages Python (Python Software Foundation) and R (R Foundation for Statistical Computing) through their network and engagement communities as common standards for programming [13].

### Current Study

The digital capabilities of NHS services in England are diverse, with ever-evolving services providing differing levels of digital capability, open-source use, and interoperability standards. Official recommendations and advice struggle to unify the NHS effectively. NHS trust application development teams play a vital role in uptake in these areas, but in the present landscape, the NHS has limited visibility of these teams and their capabilities. NHS trust application development teams are focused on the creation and deployment of digital technologies designed to improve clinical care, patient outcomes, health system efficiency, and user engagement. This includes the development of software applications for clinical workflows or patient self-management, websites that provide access to health information or services, and innovative technological solutions such as artificial intelligence tools, remote monitoring systems, or digital diagnostics that address existing challenges or create new capabilities within health care delivery. The key objective of this paper is to illuminate the current state of NHS trust application development teams in England by documenting their capabilities and technologies, while comparing standard trusts with those that have received extra funding via the GDE program, thus providing a starting point for the evaluation of current practice.

## Methods

### Design

We documented the capabilities and technologies used in the NHS to develop digital products and services. Our data source was Freedom of Information (FOI) requests sent to NHS trusts in England. In our FOIs to NHS trusts, we asked for the disclosure of all departments and teams handling application and software development, websites, and technical innovation, along with the number of team members and roles. Further information regarding the teams was requested, covering technologies and software design methodologies used, use of open source, development of service user websites and apps, innovative collaboration with clinical staff, tenders for system development, developments for others, selling systems commercially, and use of innovation grants. Eleven of the 14 FOI questions were designed to elicit clear binary responses (1=yes, 0=no), which made them straightforward to answer and reduced the potential for variability or interpretation bias across NHS trusts. Respondents could still provide additional detail if available, but the core data remained consistent (Table 1). To ensure the information received was relevant, focused, and reflective of genuine NHS digital capabilities, all trusts were appraised against the inclusion criterion of having teams and departments handling application and software development, websites, and technical innovation. To meet this criterion, the NHS trust had to respond “yes” to question 1 (Table 1). The explicit exclusion criteria were trusts without dedicated teams handling application and software development, websites, or technical innovation; trusts that did not respond or declined to provide information; and those that submitted their FOI response after December 31, 2020.

**Table 1.** Questions asked of National Health Service trusts.

Question	Wording
1	Does your trust have teams/departments that handle any of the following: application/software development, websites, or technical innovation? (Yes/No). If yes, please name these teams/departments.
2	How many members are in these teams/departments? What roles are the teams/departments made up of?
3	What technologies do the teams/departments use? For example: HTML5, C#, SQL, and .NET Core 2.0.
4	Are the developments of the teams/departments open source? (Yes/No). If yes, please provide any details you may be able to disclose regarding this.
5	What software methodology do the teams/departments use? (For example, one or more of the following: PRINCE2, AGILE, or Waterfall).
6	Do the teams/departments develop front-facing service user websites/apps? (Yes/No). If yes, please give any details you may be able to disclose regarding this.
7	Do the teams/departments work with clinical staff to formulate any innovative ideas they may have? (Yes/No). If yes, please give any details you may be able to disclose regarding this (digital innovation refers to the application of digital technology to existing business problems).
8	Have the teams/departments ever gone for external tenders for health care system developments? (Yes/No). If yes, please give any details you may be able to disclose regarding this.
9	Have the teams/departments ever done developments for other trusts/external entities? (Yes/No). If yes, please provide any details you may be able to disclose regarding this.
10	Have the teams/departments ever sold a development they produced commercially? (Yes/No). If yes, please provide any details you may be able to disclose regarding this.
11	Have the teams/departments ever attempted to secure an innovation grant? (Yes/No). If yes, please provide any details you may be able to disclose regarding this.
12	Were the teams/departments used during the COVID-19 crisis? (Yes/No). If yes, please provide any details you may be able to disclose regarding this. If no, please explain why they were not used.
13	Does your service provide mental health services? (Yes/No). If yes, have the teams/departments been involved in developing these digitally/online?
14	Do the teams/departments feel they have good visibility within the trust regarding the services they can offer? (Yes/No). Please provide any details you may be able to disclose regarding this.

All technologies were appraised against the inclusion criterion of being a programming language, framework, library, database, query language, interoperability standard, application programming interface, web service, or version control and collaboration platform. To meet this criterion, it had to be possible to find the technology via a Google search when the technology’s name was entered as the search term, and the returned results identified the technology as matching one of the inclusion criteria. Examples of matches that were deemed acceptable included legitimate sources such as developer websites, corporations, and media outlets.

### Procedure

On June 8, 2020, a list of NHS trusts in the United Kingdom was requested through an FOI email to NHS England, asking for the contact details of all NHS trusts within the United Kingdom. This yielded a list of 226 NHS trusts. During July 2020, the FOI email address and FOI contact form for each trust were identified, and an FOI email request was sent to the Freedom of Information Act (FOIA) officer or department of each of the 226 trusts. The questions asked are reported in Table 1. Each initial question had a further open-ended subquestion asking for more details when a trust confirmed the existence of any associated information. Since FOI responses vary in format and structure across NHS trusts, the data had to be interpreted on a case-by-case basis rather than through a uniform extraction template. According to the FOIA, requests must be answered within 20 working days of receipt [14]. Due to the COVID-19 pandemic, the author allowed until December 31, 2020, for a response to keep all data within the same year. Retrospective ethical approval was requested from the Aston University Department of Business and Social Sciences Ethics Committee.

### Content and Data Analysis

A conceptual content analysis methodology was used to conduct the data analysis. This approach facilitated the extraction of both quantitative and qualitative insights from the open-ended responses obtained via FOI requests. Specifically, it enabled the generation of a high-level statistical overview of digital capabilities across NHS Trusts, as well as a more granular examination of the technologies and methodologies reported by these institutions in relation to the study objectives.

To support systematic analysis, a codebook was developed to categorize the FOI responses (Textbox 1). Based on this codebook, the lead author designed and implemented a bespoke data management system through which the FOI response data were processed. Following data entry, the coded responses were exported as a complete dataset for statistical analysis using SPSS (IBM Corp).

Textbox 1. **Codebook.**

**Question 1:** Does your trust have teams/departments that handle any of the following: application/software development, websites, technical innovation?

Response options:

1. No
2. Yes
3. N/A

Number of teams: if option 1 was coded, calculate the total number of teams based on the response.

**Question 2:** How many members are in these teams/departments?

Response options:

1. Undisclosed
2. Disclosed
3. Ambiguous
4. Not applicable
5. Unanswered
6. Unheld information
7. Declined under a section of the Freedom of Information Act.

Number of team members: If 1 response 1 was coded, calculate the total number of team members based on the response.

**Question 3:** What technologies do the teams/departments use? For example: HTML5, C#, SQL, and .NET Core 2.0.

Response options:

1. Undisclosed
2. Disclosed
3. Ambiguous
4. Not applicable
5. Unanswered
6. Unheld information
7. Declined under a section of the Freedom of Information Act.

Technologies and technology categories: if response 1 was coded, list all technologies in the response and code each technology against one of the following categories:

- Programming language: a language used by a programmer to communicate with computers. They are mainly used to develop desktop, website, and mobile apps.
- Framework: a structure upon which software can be built. They serve as a foundation, so a programmer does not have to start entirely from scratch every time they write a new program.
- Library: a collection of prewritten code that programmers can use to optimize tasks.
- Database: an organized collection of structured information, typically stored electronically in a computer system.
- Query language: a language used to make queries in databases and information systems.
- Interoperability standard: a standard that enables the operational processes, underlying exchange, and sharing of information between different systems.
- API or web service: a technology that enables the transfer of data between separate software applications.
- Version control and collaboration platforms: facilitate coordination, sharing, and collaboration across the entire software development team. Version control software enables teams to work in distributed and asynchronous environments, manage changes and versions of code and artifacts, and resolve merge conflicts and related anomalies.
- Other

**Question 4:** Are the developments of the teams/departments open source? (Yes/No). If yes, please give any details you may be able to disclose regarding this.

Response options:

1. No
2. Yes
3. Sometimes
4. Ambiguous
5. Not applicable
6. Unanswered
7. Unheld information
8. Declined under a section of the Freedom of Information Act.

**Question 5:** What software methodology (eg, PRINCE2, AGILE, or Waterfall) do the teams/departments use?

Response options:

1. Undisclosed
2. Disclosed
3. Ambiguous
4. Not applicable
5. Unanswered
6. Unheld information
7. Declined under a section of the Freedom of Information Act.

Software design methodologies: if response 1 was coded, all methodologies should be appraised and coded against the inclusion criterion of being a software design methodology.

**Question 6:** Do the teams/departments develop front-facing service user websites/apps? (Yes/No). If yes, please give any details you may be able to disclose regarding this.

Response options:

1. No
2. Yes
3. Ambiguous
4. Not applicable
5. Unanswered
6. Unheld information
7. Declined under a section of the Freedom of Information Act.

**Question 7:** Do the teams/departments work with clinical staff to formulate any innovative ideas they may have? (Yes/No). If yes, please give any details you may be able to disclose regarding this.

Response options:

1. No
2. Yes
3. Ambiguous
4. Not applicable
5. Unanswered
6. Unheld information
7. Declined under a section of the Freedom of Information Act.

**Question 8:** Have teams and departments ever done developments for other trusts or external entities?

Response options:

1. No
2. Yes
3. Ambiguous
4. Not applicable
5. Unanswered
6. Unheld information
7. Declined under a section of the Freedom of Information Act.

**Questions 9:** Have teams and departments ever attempted to secure an innovation grant?

Response options:

1. No
2. Yes
3. Ambiguous
4. Not applicable
5. Unanswered
6. Unheld information
7. Declined under a section of the Freedom of Information Act.

A detailed classification of the types of data extracted from the FOI responses is provided in Table 2, as categorized using the codebook. The dataset predominantly consisted of dichotomous variables.

**Table 2.** Collected data types from content analysis.

Question	Collected data types
1	Yes or no (dichotomous) Number of teams (numerical) Details (qualitative)
2	Number of members (numerical) Details (qualitative)
3	Technology names (nominal) Technology categories (nominal) Technology companies (nominal) Uses legacy tech (dichotomous) Details (qualitative)
4	Yes or no (dichotomous)
5	Methodology names (nominal) Details (qualitative)
6	Yes or no (dichotomous) Details (qualitative)
7	Yes or no (dichotomous) Details (qualitative)
8	Yes or no (dichotomous) Details (qualitative)
9	Yes or no (dichotomous) Details (qualitative)

To establish whether the answers to these questions differed as a function of trust funding (standard or GDE), tests of association were used. A series of chi-square tests was conducted as an additional layer of validation to enhance the robustness of the findings for questions 1, 3, 4, 6, 7, 9, and 11. For analysis, “No” and “N/A” responses were collapsed into a single category, and only “Yes” and “No” responses were included in the final tests. Assumptions were checked, and when violated, a Fisher extract was used instead. All analyses were conducted using SPSS for Windows (version 25; IBM Corp).

### Ethical Considerations

The Aston University Department of Business and Social Sciences Ethics Committee has reviewed and granted approval for the study. The study used UK FOIA data from NHS trusts, a process that supports transparency and public access to government-held information. The ethical considerations primarily focused on ensuring adherence to FOIA guidelines and that the requests were legitimate, appropriate, and respectful of the privacy rights of individuals and NHS trusts involved.

As the study did not involve human participants directly, and the data requested were publicly available, there were no concerns regarding private or sensitive data. Ethical issues related to informed consent and participant confidentiality were not applicable. However, all data requests were made in alignment with the principles of transparency, fairness, and public accountability set out by the FOIA.

In addition, the study adhered to best practices for data handling and reporting, ensuring that the requested data were used responsibly and in compliance with data protection laws where applicable. Any personal or sensitive information was either excluded or anonymized to protect confidentiality. To ensure fairness and safeguard the identity of NHS trusts, all reporting was done in a manner that made it impossible to trace data back to individual organizations, unless the FOI responses were requested directly from the NHS Trusts.

## Results

### Overview

We present the data pertaining to (1) response rates, (2) capabilities, (3) technologies, (4) open source, and (5) interoperability standards.

### Response Rates

Out of 226 trusts, 191 (84.5%) completed the FOI request, 23 (10.2%) acknowledged receiving the FOI request but never provided followed up with answers, 7 (3.1%) had merged with another NHS trust, 2 (0.9%) sent partial information consisting of an organizational plan, 2 (0.9%) refused the FOI because it would take too long to complete, and 1 (0.4%) FOI email address no longer accepted emails. In total, 161 (84.3%) met the inclusion criteria by having teams or departments handling application and software development, websites, and technical innovation. The final sample comprised 83% (134/161) standard trusts and 17% (27/161) GDE trusts.

### Capabilities

#### Have Software and App Development, Website, and Innovation Teams

Overall, 161 out of 191 (84%) trusts said they had software and app development, website, and innovation teams. The chi-square assumptions were marginally violated, with 25% of the cells (1 out of 5) having an expected count of less than 5; the minimum expected count was 4.87, so a Fisher exact test was run. According to the Fisher exact test, there was a nonsignificant association between trust type and having teams (*P*=.79). This suggests no difference in team presence between standard and GDE trusts.

#### Developing Front-Facing Service User Websites and Apps

Overall, 112 out of 161 (69.6%) trust teams developed front-facing service user websites and apps. A total of 43 (26.7%) trusts said no, 3 (1.9%) said the question was not applicable, 1 (0.6%) declined to answer the question on grounds of security, 1 (0.6%) stated the information was uncaptured, and 1 (0.6%) did not answer the question. There was a nonsignificant association between trust type and developing front-facing service user websites and apps (*χ*^2^_1_=1.47; *P*=.23). This suggests no difference between standard and GDE trusts.

#### Work With Clinical Staff to Formulate Innovative Ideas

Overall, 150 out of 161 (93.2%) of trust teams work with clinical staff to formulate innovative ideas. A total of 8 (5%) trusts said no, 2 (1.2%) said the question was not applicable, and 1 (0.6%) stated the information was uncaptured. The chi-square assumptions were marginally violated, with 25% of the cells having expected counts less than 5, and the minimum expected count was 1.69, so a Fisher exact test was run. According to the test, there was a nonsignificant association between trust type and the work with clinical staff to formulate innovative ideas (*P*=.21). This suggests no difference between standard and GDE trusts.

#### Carried Out Developments for Other Trusts and External Entities

Overall, 55 out of 161 (34.2%) trust teams carry out developments for other trusts or external entities. One hundred (62.1%) of trusts said no, 3 (1.9%) said the question was not applicable, 1 (0.6%) declined to answer the question on grounds of security, 1 (0.6%) stated the information was uncaptured, and 1 (0.6%) did not answer the question. There was a nonsignificant association between trust type and carrying out developments for other trusts and external entities (*χ*^2^_1_=0.031; *P*=.86). This suggests no difference between standard and GDE trusts.

#### Attempted to Secure an Innovation Grant

Overall, 35 out of 161 (21.7%) trust teams have attempted to secure an innovation grant. A total of 118 (73.3%) trusts said no, 3 (1.9%) said the question was not applicable, 4 (2.5%) stated the information was uncaptured, and 1 (0.6) declined to answer on the grounds of security. There was a nonsignificant association between trust type and attempting to secure an innovation grant (*χ*^2^_1_=1.57; *P*=.21). This suggests no difference in attempts to secure innovation grants between standard and GDE trusts.

### Technologies

Overall, 138 out of 161 (86%) trusts with teams disclosed the technologies they used. A total of 84 technologies were identified: 27 programming languages, 19 frameworks, 10 libraries, 11 databases, 1 query language, 7 interoperability standards, 5 application programming interfaces or web services, and 4 version control and collaboration platforms. All technologies are summarized in Table 3.

**Table 3.** All technologies reported to be used by the National Health Service for digital development.

Category and technology	Developer or organization	NHS^a^ trusts using (N=138), n (%)
Query language		
SQL^b^	ISO^c^	88 (63.8%)
Programming languages		
C#	Microsoft	81 (58.7%)
JavaScript	ECMA^d^ International	54 (39.1%)
PHP^e^	PHP Group	20 (14.5%)
VB.NET^f^	Microsoft	15 (10.9%)
VB^g^	Microsoft	14 (10.1%)
Java	Oracle Corporation	10 (7.2%)
Python	Python Software Foundation	7 (5.1%)
VBA^h^	Microsoft	4 (2.9%)
Delphi	Embarcadero Technologies, Incorporated	3 (2.2%)
C++	ISO and IEC^i^	3 (2.2%)
Ruby	Open Standard	3 (2.2%)
CFML^j^	Adobe	2 (1.4%)
XSLT^l^	W3C^k^	2 (1.4%)
R	R Core Team	2 (1.4%)
ObjectScript	InterSystems	2 (1.4%)
PowerShell	Microsoft	2 (1.4%)
Oracle PL/SQL^m^	Oracle Corporation	2 (1.4%)
TypeScript	Microsoft	1 (0.7%)
Meditech Magic	Medical Info Tech, Incorporated	1 (0.7%)
ASP.NET^n^ Razor	Microsoft	1 (0.7%)
C	ANSI^o^	1 (0.7%)
ECMAScript^p^	ECMA International	1 (0.7%)
XAML^q^	Microsoft	1 (0.7%)
Elixir	Plataformatec	1 (0.7%)
VB 6	Microsoft	1 (0.7%)
Lua	Tecgraf^r^	1 (0.7%)
Perl	Larry Wall	1 (0.7%)
Frameworks		
.NET	Microsoft	42 (30.4%)
.NET Core	Microsoft	40 (29.0%)
ASP.NET MVC^s^	Microsoft	22 (15.9%)
ASP.NET	Microsoft	21 (15.2%)
Classic ASP^t^	Microsoft	8 (5.8%)
Angular	Google	8 (5.8%)
Entity Framework	Microsoft	5 (3.6%)
Xamarin	Microsoft	5 (3.6%)
ASP.NET WebForms	Microsoft	4 (2.9%)
AngularJS	Google	3 (2.2%)
Vue.js	Evan You	2 (1.4%)
Ionic	Drifty	2 (1.4%)
WPF^u^	Microsoft	2 (1.4%)
Blazor	Microsoft	1 (0.7%)
Symfony	Symfony community	1 (0.7%)
Hibernate	Red Hat Software	1 (0.7%)
LINQ^v^	Microsoft	1 (0.7%)
Spring	VMware	1 (0.7%)
Oracle Forms	Oracle Corporation	1 (0.7%)
Libraries		
jQuery	The jQuery Team	21 (15.2%)
React	Meta and community	4 (2.9%)
AJAX^w^	Adaptive Path	3 (2.2%)
Knockout.js	Knockout	1 (0.7%)
SignalR	Microsoft	1 (0.7%)
SweetAlert	Tristan Edwards	1 (0.7%)
TensaFlow	Google Brain Team	1 (0.7%)
chart.js	Chart.js Team	1 (0.7%)
jQuery UI^x^	The jQuery Team	1 (0.7%)
WinForms	Microsoft	1 (0.7%)
Databases		
Microsoft SQLServerr	Microsoft	29 (21.0%)
Caché and Ensemble	InterSystems	12 (8.7%)
MySQL^y^	Oracle Corporation	4 (2.9%)
PostgreSQL	PostgreSQL Global Group	4 (2.9%)
Oracle	Oracle Corporation	3 (2.2%)
PouchDB	Apache Software Foundation	1 (0.7%)
MariaDB	MariaDB Foundation	1 (0.7%)
MongoDB	MongoDB, Incorporated	1 (0.7%)
CouchDB	Apache Software Foundation	1 (0.7%)
SQlite	Richard Hipp	1 (0.7%)
NoSQL	Unidentifiable	1 (0.7%)
Interoperability		
HL7^z^	HL7 International	11 (8.0%)
JSON^aa^	ECMA International	10 (7.2%)
XML^ab^	W3C	7 (5.1%)
Arden Syntax	HL7 International	3 (2.2%)
Mirth Connect	NextGen Healthcare	3 (2.2%)
FHIR^ac^	HL7 International	3 (2.2%)
NextGen Connect	NextGen Healthcare	1 (0.7%)
APIs^ad^ and web services		
Web Services	Unidentifiable	3 (2.2%)
RESTful APIs	Unidentifiable	3 (2.2%)
WebAPI^ae^	Microsoft	3 (2.2%)
SOAP^af^ XML Web services	Unidentifiable	2 (1.4%)
APIs	Unidentifiable	1 (0.7%)
Version control and collaboration platform		
Git	Junio Haman	5 (3.6%)
Azure DevOps^ag^	Microsoft	4 (2.9%)
TFS^ah^	Microsoft	2 (1.4%)
GitLab	GitLab Incorporated	1 (0.7%)

a NHS: National Health Service.

b SQL: Structured Query Language.

c ISO: International Organization for Standardization.

d ECMA: European Computer Manufacturers Association.

e PHP: Hypertext Preprocessor.

f VB.NET: Visual Basic .NET.

g VB: Visual Basic.

h VBA: Visual Basic for Applications.

i IEC: International Electrotechnical Commission.

j CFML: ColdFusion Markup Language.

k W3C: World Wide Web Consortium.

l XSLT: Extensible Stylesheet Language Transformations.

m PL/SQL: Procedural Language extensions to the Structured Query Language.

n ASP.NET: Active Server Pages .NET.

o ANSI: American National Standards Institute.

p ECMAScript: European Computer Manufacturers Association Script.

q XAML: Extensible Application Markup Language.

r Tecgraf: Computer Graphics Technology Group.

s MVC: model–view–controller.

t ASP: Active Server Pages.

u WPF: Windows Presentation Foundation.

v LINQ: Language-Integrated Query.

w AJAX: Asynchronous JavaScript and XML.

x UI: user interface.

y MySQL: My Structured Query Language.

z HL7: Health Level Seven.

aa JSON: JavaScript Object Notation.

ab XML: Extensible Markup Language.

ac FHIR: Fast Healthcare Interoperability Resources.

ad API: Application Programming Interface.

ae WebAPI: Web Application Programming Interface.

af SOAP: Simple Object Access Protocol.

ag DevOps: Development and Operations.

ah TFS: Team Foundation Server.

Python, the programming language used and promoted by NHS England and the NHS Transformation Directorate, was disclosed by 7 out of 138 (5.1%) trusts with teams that disclosed technologies. R, the programming language used for statistics and data science, was disclosed by 2 out of 138 (1.4%) trusts with teams that disclosed technologies. The most disclosed query language was Structured Query Language with 88 out of 138 (63.8%) trusts, and the most disclosed programming language was C# with 81 out of 138 (58.7%) trusts.

### Open Source

Overall, 25 out of 138 (15.5%) trust teams said they always used open-source technology, and 9 out of 138 (5.6%) said they used open-source sometimes. In total, 119 (73.9%) trusts said no, 6 (3.7%) said the question was not applicable, and 2 (1.2%) declined to answer on the grounds of security. There was a nonsignificant association between team type and open-source use (*χ*^2^_1_=0.014; *P*=.91). This suggests no difference in the use of open source between standard and GDE teams. Eight out of 138 (5.8%) trusts with teams that disclosed technologies indicated they used version control and collaboration platforms. A total of 4 platforms were identified in this review. The most used was Git, reported by 5 out of 138 (3.6%) trusts with teams that disclosed technologies.

### Interoperability Standards

Overall, 24 out of 138 (17%) trusts with teams that disclosed technologies referred to interoperability standards in their responses. A total of 7 interoperability standards were identified. The most disclosed interoperability technology was HL7, mentioned by 11 out of 138 (8%) trusts.

## Discussion

### Principal Findings

This study is the first attempt to document the capabilities and technologies of NHS trust application development teams at a particular point in time. While these capabilities and technologies are changeable, the paper provides future researchers, commissioners, and policy makers with a baseline from which to build. The data presented raise several interesting questions relating to capabilities, open-source use, interoperability use, and programming languages.

### Data Accessibility, Quality, and Limitations

The study was reliant on the provisions of the UK FOIA (2000) for data collection. Approximately 85% of NHS trusts responded to the request. Although this represents a large sample of trusts, it is difficult to ascertain the extent to which they are representative of all trusts. FOI requests rely on the expertise of those responsible for handling them in any given NHS trust, and the author was unable to determine how accurate and thorough individual responses were. During the data collection period, trusts were under immense pressure due to the COVID-19 pandemic, meaning that many may not have had the capacity to respond accurately. While most trusts addressed the questions posed, some declined to respond on security grounds. These data gaps, however, were minimal.

### Capabilities

Digital maturity across NHS trusts is presently considered to be underdeveloped, with previous reports highlighting the need for the NHS to bridge the digital divide to avoid the creation of a 2-tier secondary care system [10]. While this may still be true in some cases, the data presented here highlights that in-house processes to develop digital capability are well established in 84% of responding trusts. Overall, no statistical differences were found between trust types and these results. This may indicate that increasing in-house capability to carry out digital projects was not a consideration when trusts received GDE funding. These results also highlight an issue with digital maturity measures such as HIMSS EMRAM (Healthcare Information and Management Systems Society: Electronic Medical Record Adoption Model). The measure exclusively focuses on technological functionality, such as electronic health records, and does not consider human and organizational capabilities [2]. The apps lead to an incomplete picture of a trust’s digital maturity. While no significant differences were found between trust types, the overall results for each question highlight some interesting findings.

A review by Sood and McNeil [15] highlighted that it would be impossible for the NHS to become a modern, effective, and efficient health care system without fully embracing the digital agenda. The results of this survey reveal that trusts are working toward overcoming Sood and McNeil’s [15] concern, with 69.6% of respondents reporting the creation of websites and apps to help service users.

A study by Asthana et al [16] investigated how, despite having a tax-funded NHS and a strong policy toward eHealth innovations, the English NHS had been limited in the extent to which it exploited digital potential. The survey results indicate that 93% (150/161) of respondents help clinical staff formulate innovative ideas. So, despite the suggestion of limited exploitation, NHS trusts are actively working to enable their clinical staff to innovate. However, the data do not reveal why, if this practice is commonplace, it has not been exploited to its full potential. Possible reasons may include limited resources regarding time, staffing, and funding.

The NHS Global Exemplar program was implemented to establish a knowledge-sharing ecosystem to spread learning among NHS trusts [2]. The survey results indicate 34% (55/161) carry out this practice. NHS England needs to do more to promote digital collaboration between trusts. Encouraging more digital development between organizations would help innovation and the leveling up of digital NHS services. Making trust teams aware of collaboration platforms such as the FutureNHS, that is used to support the right technology solutions to health and social care [17], could enable better sharing of technical innovations between trusts.

Innovation is critical in NHS England achieving the ambitions set out in the mandate, to increase the pace and scale of change. The NHS’s Accelerated Access Collaborative (AAC) reported 2708 innovations being developed in 2019/2020 [18]. Survey data indicate that only 21.7% of respondents had applied for innovation grants. NHS England needs to do more to promote the innovation funding available to trusts. Academic Health Science Networks (AHSNs), now known as the Health Innovation Network, were established by NHS England in 2013 to spread innovation, improve health outcomes, and generate economic growth [19]. If the Health Innovation Network were made more accessible to trust development teams, the internal potential of trusts to unlock innovation could help to maximize their benefit and better fund digital maturity. In addition, trusts could use the funding streams advised by the Health Innovation Network to independently create new digital innovations. Unfortunately, there is a limited amount of investment available, and not all trusts can benefit from this type of funding, but more should be done to encourage trusts to apply for these grants.

### Open-Source Technology

NHS England has tried to instill open-source technology within the NHS for nearly a decade through multiple strategic programs such as the Open-Source Program and Code4Health. The latter aimed to serve as a vessel for promoting open-source technology through clinicians [20]. The survey results revealed that 73.9% of trust development teams do not use open-source approaches, suggesting that previous initiatives have had limited cultural influence, with developers still opting for closed-source methods. Recent publications, such as the report by Goldacre et al [21], have helped to put forward proposals for using open-source implementation and draw focus to NHS data analysts.

GitHub is the NHS’s preferred platform for publishing open-source code [13]. However, the results of the survey show that only 5.8% of respondents listed using version control and collaboration platforms, and none of these gave reference to GitHub. NHS England needs to better communicate its desire for the NHS to use GitHub and open source and to work to promote this across all services. Without a unified approach to implementation, GitHub and open-source practices will continue to be used inconsistently across the NHS. The NHS data strategy has committed to making all new source code that is produced or commissioned open and reusable by default [1]. For this commitment to succeed in the NHS, the DHSC needs to go beyond publication of policy and digital guidance. Training and tools will be required to enable this at a regional level, meaning significant investment would be required, as services currently do not have the funding capacity.

There are presently 3 network and engagement communities run by NHS England’s Transformation Directorate: Analyst X, NHS-R, and NHS Python [22]. The NHS Python community is focused on the promotion of open-source practices. However, this is done purely in conjunction with the Python programming language. Survey results showed that only 5.1% of respondents disclosed using Python. For open-source to be more widely adopted, a new open-source program of activity is required for the NHS. This should be aimed at the full spectrum of roles within the data process cycle and focus on dominant technologies used by NHS services. This, in turn, will make the community more inclusive to all NHS services and help to increase the adoption of open-source technology. The program should be coordinated at a regional level, with open-source champions that have both technical knowledge and understanding of data processing at the helm. The actions required to carry out this recommendation would be a large undertaking, and whether NHS England has the time or capacity to do this in the current climate should be considered; it may not be feasible. It will also be reliant on trusts being willing to involve themselves in the program.

### Interoperability Standards

The objective to improve interoperability has been included in several NHS plans, and the end benefits of advanced interoperability are well recognized [23]. The primary benefit of interoperability is the ability to offer safe and reliable information transfer across the care pathway, reducing the possibility of adverse clinical outcomes [24]. The survey showed 17% listed interoperability standards in the technologies they disclosed. The DHSC has emphasized that it wants the NHS to use Fast Healthcare Interoperability Resources (FHIR) to achieve interoperability (DHSC, 2020). Unfortunately, the most disclosed standard was HL7, the predecessor to FHIR, disclosed by 8% of trust teams. Better promotion of the NHS Digital Interoperability Toolkit would benefit NHS trusts by helping them to standardize their technology and interoperability specifications [25]. Uptake, however, would be reliant on trust and willingness to use the toolkit.

### Technologies

Interoperability is reliant on the NHS using technologies that are capable of communicating with one another. The most used programming language named in the survey was C# with 58.7%, a language that differs from NHS England’s preferred programming language of Python, which was disclosed by 5.1% of those in the study. The difference in programming language standards puts the Transformation Directorate’s push for open source and code sharing at odds with the wider NHS. There is still a benefit to code sharing, but some of this is lost because of the overhead involved in migrating between programming languages. A focus on the most consistently used languages across the NHS services would help to unify the NHS. There will be a strong reliance on the languages they have become accustomed to, and moving away from these is difficult to implement. Any coding examples NHS England develops should be written in the programming languages most appropriate for NHS services. Ensuring the use of modern languages will enable the NHS to keep a competitive advantage and help to retain staff looking to work on more relevant and modern languages over time.

### Recommendations

Some of the insights derived from the study and related discussion suggest that the NHS has a solid foundation in certain areas. While trusts may not yet be fully aligned in their use of shared code, source control systems, or in leveraging all available opportunities, such as relevant grants, there is clear evidence of technology utilization across the organization. To build upon these foundations, this paper offers the following recommendations as steps toward achieving a more comprehensive digital maturity.

#### Promoting Digital Collaboration

Enhancing digital development between organizations can foster innovation and elevate digital NHS services. By making trust development teams aware of collaborations, such as the FutureNHS platform, we can enhance the sharing of technical innovations across trusts.

#### Leveraging the Health Innovation Network for Innovation

Increasing the accessibility of the Health Innovation Network to trusted development teams could unlock significant internal innovation potential, aiding in the funding and advancement of digital maturity. Trusts could independently develop new digital innovations by using funding streams advised by the Health Innovation Network.

#### Promoting Open-Source Usage

NHS England should better communicate its advocacy for using GitHub and open-source technologies and actively promote this initiative across all services. The NHS Data Strategy aims to make all new source code open and reusable by default. However, to achieve this, in addition to publishing policy and digital guidance, substantial investment in training and tools is required at a regional level, as current services lack sufficient funding capacity.

#### Increasing Open-Source Adoption

The survey revealed that only 5.1% of respondents use Python, indicating a need for a comprehensive open-source program across the NHS. This program should target all roles within the data process cycle and focus on the prevalent technologies used in NHS services. By making the community more inclusive, the adoption of open-source technology can be increased. The program should be regionally coordinated, with open-source champions possessing both technical expertise and an understanding of data processing leading the initiative.

#### Aligning Programming Language Standards

The variation in programming language standards presents a challenge to the Transformation Directorate’s push for open source and code sharing across the NHS. Although there are benefits to code sharing, the overhead involved in migrating between different programming languages diminishes some of these benefits. Focusing on the most widely used languages across NHS services could unify efforts. It is essential to ensure that coding examples provided by NHS England are developed in languages most appropriate for NHS services, thereby maintaining a competitive advantage and retaining staff interested in working with modern technologies.

### Future Research Needs

Future research should analyze digital innovation across NHS trusts, focusing on barriers such as limited resources, organizational culture, and technical expertise. Understanding these challenges is crucial for creating strategies to reduce disparities and promote equitable progress. Research should also explore ways to increase the adoption of open-source technologies, evaluating programs such as NHS Python and Analyst X, and expanding their reach. Investigating the programming languages and digital tools used across NHS trusts, aligned with NHS England’s standards, can enhance interoperability and improve code sharing. In addition, assessing the feasibility and impact of unified programming standards within the NHS’s digital infrastructure is essential. Research in these areas could significantly accelerate the NHS’s digital transformation, ensuring services remain efficient, innovative, and inclusive, thereby improving health care delivery and stakeholder satisfaction.

### Conclusion

Interoperability principles enable the use of data and information across system and organizational boundaries. Embracing interoperability addresses the unrealistic expectation for a one-size-fits-all system for the NHS, or that heterogeneous systems will communicate with one another out of the box. Interoperability reduces the need for greater oversight across the spectrum and allows the NHS to demonstrate maturity at crucial sinews and joints between systems and services.

Without interoperability, much-needed digital maturity will not be gained, and the divide between NHS trusts with and without GDE funding will continue to grow. As the NHS continues to advance its plans for the use of digital, open-source technology and interoperability standards, it is essential that a unified approach is followed. This study has provided a snapshot of the digital development technology landscape and the areas of digital implementation policy that need the most refinement to improve their success.

## Abbreviations

AAC: Accelerated Access Collaborative
AHSN: Academic Health Science Network
DHSC: Department of Health and Social Care
FAIR: findability, accessibility, interoperability, and reusability
FHIR: Fast Healthcare Interoperability Resources
FOI: Freedom of Information
FOIA: Freedom of Information Act
GDE: Global Digital Exemplar
HIMSS EMRAM: Healthcare Information and Management Systems Society: Electronic Medical Record Adoption Model
NHS: National Health Service
SQL: structured query language
UKHSA: UK Health Security Agency
